# Between personal and relational privacy: understanding the work of informed consent in cancer genetics in Brazil

**DOI:** 10.1007/s12687-015-0234-4

**Published:** 2015-05-22

**Authors:** José Roberto Goldim, Sahra Gibbon

**Affiliations:** Bioethics Research Laboratory, Hospital de Clínicas de Porto Alegre, Universidade Federal do Rio Grande do Sul, Porto Alegre, Brazil; Medical School Pontifícia Universidade Católica do Rio Grande do Sul, Universidade Federal do Rio Grande do Sul, Porto Alegre, Brazil; Anthropology Department, University College London, London, UK

**Keywords:** Bioethics, Privacy, Genetic counselling, Cancer, Informed consent

## Abstract

Drawing from perspectives of both bioethics and anthropology, this article explores how the boundaries between personal and relational privacy are negotiated by patients and practitioners in the context of an emerging domain of cancer genetics in Brazil. It reflects on the place of informed consent in the history of bioethics in North America in contrast to the development of bioethics in Brazil and the particular social cultural context in which consent is sought in Brazilian public health care. Making use of empirical research with families and individuals receiving genetic counselling related to increased genetic risk for cancer, in genetic clinics in southern Brazil, it examines how informed consent is linked to the necessary movement between personal and relational privacy. The paper illustrates the value of a particular tool known as a ‘sociogram’ to examine the complex interpersonal dynamics that arise in negotiating informed consent at the interface between the family and the individual in Brazil. The paper, therefore, points to the scope of further interdisciplinary exchanges between anthropology and bioethics, confronting the new challenges that arise in the context of medical genetics in developing country.

## Introduction

Two issues are particularly relevant when considering the specific bioethical challenges raised by developments in genetic medicine: informed consent and privacy.

The first concerns the challenges of informed consent given the way that much medical genetics operates at a boundary, often difficult to separate, between health care and research (Hallowell 2009; Hallowell et al. 2010). In regular health care contexts, the patient brings a medical need to be evaluated and treated by professionals, while in a research scenario, the researcher offers a possibility of participation in a project. In genetic medicine, health care necessities are entangled with research possibilities and potentials. This may create problems for professionals and patients/participants with respect to issues such as consent. Patients/participants may find it difficult to understand the difference between research and health as separate fields of activity (Bosk 2002). These issues may be particularly acute in developing and low and middle income country contexts, such as in Latin American, where public health is precarious for many people in ways that often make participation in research a means of accessing basic health care resources. This is an expression of ‘ethical variability’ of institutional or transnational research cultures, particularly in the context of the outsourcing of clinical trial research (Petryna 2009).

The second issue relates to privacy. The most common way to understand privacy and consent is at the individual level. Developments in medical genetics extend these concepts beyond the individual, because related persons may become involved, as they are themselves at genetic risk or in a situation to benefit from familial information about potential genetic risk. In this new context, protection of personal sensitive data takes on a new (expanded) meaning of relational privacy (Ursin 2008). As a result, informed consent may only be achieved when addressed within the wider scope of the family (Hallowell 1999).

This paper makes use of approaches within the disciplines of bioethics and anthropology to explore the challenges of informed consent and privacy in the context of the emerging domain of cancer genetics in Brazil. It pays particular attention to how the boundaries between personal and relational privacy are negotiated by patients and practitioners. First, it reflects on the place of informed consent in the history of bioethics in North America, in contrast to the development of bioethics in Brazil; before outlining the particular social cultural context in which consent is sought in Brazilian public health care settings. Making use of empirical research with families and individuals receiving genetic counselling related to genetic risk for cancer in genetic clinics in southern Brazil, it illustrates how informed consent is linked to the necessary movement between personal and relational privacy. This is further explored in relation to the use of a particular tool, known as a ‘sociogram’, to examine the complex interpersonal dynamics that arise when negotiating informed consent at the interface between the family and the individual in Brazil.

## The historical evolution of informed consent in North America and beyond

The theoretical basis for the informed consent rationale has emerged, especially in the USA, on a contractualist basis (Beauchamp and Childress 1978). In that context, the professional has a duty to inform and to respect the voluntariness of another capable person. This person, after being properly informed, chooses to accept or reject the offer made to him/her, exercising his/her autonomy. In this principialist perspective, the relationship between the professional and the other person involved is based on duties (Faden and Beauchamp 1986).

Seen in this way, the consent process has certain preconditions. These include the ability to understand and decide voluntarily. The process also comprises two components: information and consent itself, as in (for example) an authorization for the proposed action (Beauchamp and Faden 1995). The professional has a duty to inform while the other person involved must decide whether to allow the proposed action. Signing of the informed consent form would be understood as the proof that the process was properly undertaken, based on the assumption that the person was able to understand and decide, after being properly informed.

Many projects have been conducted to study the relationship between the capacity to provide informed consent, age and level of education. Studies have shown that it is not only adults who are capable of doing so but also teenagers and the elderly are able to decide what is in their best interest. The legal standard for capacity should not be confused with the ability in itself, which is dependent on psychological and moral development (Raymundo and Goldim 2008). Similarly, it is notable that many research participants, almost 50 %, sign a consent form without having adequate understanding of what is being proposed (Goldim et al. 2007).

Nevertheless, from within the social sciences, there has been a strong critique of the principle of informed consent and assumed personal autonomy. In North America, informed consent has been critiqued as an ‘ethical panacea’, in attemping to confront the so-called paternalism of medicine (Wolpe 1998). It has been argued that this serves to reify the process of consent and reduce it to a model of rational individual choice. As Fox and Swazey (1984) pointed out, ‘informed consent is premised on an autonomous individual, with little or no concept of social aspects’. For Corrigan, this is an ‘empty ethics’ that ‘strips the practice of consent away from social context’ (2003).

Other questions also arise for social scientists who have examined more closely the social and empirical contexts in Europe and North America, which inform the practice and process of medical research and informed consent and its assumed ‘voluntariness’. Here, the constitutive role of a range of social relations and contexts is highlighted. On the one hand, there are various interpersonal relations, such as that between patient and doctor, or the patient and the family or community, which may influence the process of obtaining informed consent (Corrigan et al. 2009). On the other hand, institutional arrangements may also impinge on and inform the process of decision-making related to medical interventions. As Høyer and Lynöe’s work demonstrates, questions of ‘trust’ in state or national institutions linked to public health care may be central to decisions to participate in medical research (2006), showing how questions of individual ‘choice’ and ‘social context’ are complexly intertwined. From this perspective, the process of obtaining informed consent is directly linked to the institutional ‘habitus’ (Bourdieu 1984) of health care practices and never a direct or uniform outcome of the deliberation of individuals.

Another key aspect that has been central to social science critique and its engagement with bioethics has been the notion that subjectivity and personhood cannot be understood in any singular way but must instead be addressed in its diversity as ‘porous, partial and malleable’ (Biehl et al. 2007).

Yet, there are numerous other bioethical approaches outside of the dominant North American paradigm that reveal an alternative history of bioethics and way of understanding the process of informed consent and autonomy, upon which the practice of bioethics in Brazil directly draws (Clotet 1995; Goldim 2002). These maybe particularly relevant when examining the issues of informed consent and privacy raised by developments in medical genetics in Brazil and beyond.

One of these alternative approaches which has also informed Brazilian bioethics concerns ‘Virtue Ethics’ (Aristotle 1999). Here, the consent process can be explained in several ways but mainly by virtue of ‘good faith, fidelity and justice’ for all participants. People involved have a commitment to have a genuine interest in participating, a duty to undertake the actions to which they are committed and, especially, not to discriminate against people due to their characteristics (Pellegrino and Thomasma 1993).

Another possibility for understanding the consent process can be based on what is described as the ‘Ethics of Intentions’, in fact first proposed in the middle ages (Abelard 1995). From this perspective, the proposal to carry out an action is assessed in relation to the agent’s intention, as well as the validity of the consent given by the person who will perform or undergo that action. The evaluation of the agent does not only need to meet the requirement to inform but also the intention associated with the proposed action to be taken. The validity of the consent depends on the knowledge and freedom associated with the decision. The ethical value associated with this process is the result of this set of related actions: agent intent and validity of the associated consent (Abelard 1144 (translated 1995)). It is important to note that this type of approach has also been historically incorporated with a North American model of informed consent (Beauchamp 2000).

The framework of ‘otherness’, proposed by Lévinas (1991), entails the recognition that consent is a process that operates within relations of responsibility, which also has influenced the development of Brazilian bioethics. In this understanding, the relationship between people results from their effective interaction, with the recognition that establishes an ethical co-presence between them. From this perspective, the consent process is effectively understood as an interaction between two or more persons. As such, there is no possibility that any participant may be neutral facing the other, as this interaction generates the responsibility inherent to the recognition of the other’s presence in a relationship (Lévinas 1991).

Finally, an Ethics of Responsibility approach (Jonas 1969) pervades the approaches already outlined, giving meaning and significance to the consent process. This reinforces the notion of vulnerability, demanding an additional protection for the person or community identified as vulnerable. The Ethics of Responsibility incorporates the perspective of ‘otherness’ outlined by Levinas, not only focusing on the responsibility for components of the consent but also reflecting upon and engaging with responsibilities for those involved in the entire process (Jonas 1969).

Latin American countries have been seen as particularly vulnerable to paternalism by the medical profession (Florencia and Salles 2006), especially in the context of informed consent (Luna 1995). The contemporary Latin American perspective of bioethics outlined here takes some of these alternative approaches that do not assume or simply champion individual autonomy as a solution in approaching the issue of consent. In this way, it moves beyond the issue of autonomy, already assuming and engaging with an interdependent perspective and, thereby, challenging a one-dimensional critique of paternalism. As we explore below, in the context of medical genetics in Brazil, consent is not simply about signing a document but a process of interaction that involves multiple parties. It is not simply a relationship of reciprocity but one of interdependence (Goldim 2002).

## Medical genetics and the question of privacy

As noted in the “Introduction”, recent developments in genetic medicine have raised new questions and ethical challenges related to informed consent. As genetic medicine expands and evolves across diverse social and cultural contexts, the complexities of genetic research related to questions of privacy, consent and the collection and storage of genetic information have been revealed.

One challenge occurs in the context of storage of biological materials where a notion of relational privacy becomes essential (Beier et al. 2011). Even supposedly anonymously maintained databases may when analysed together with other information allow the identification of persons that originally consented to the storage of their biological materials (Editorial Nature 2013).

More immediately, medical genetics raises questions about the social context of informed consent, in part because genetic information has consequences not just only for the individual but also for related others as well. Who is the ‘patient’, in the context of medical genetics, is often a complex issue. This raises ethical questions about the responsibility of the patient not only to related others and kin but also to medical professionals balancing the need to share information about possible genetic risk to other members of the family with the need to protect privacy and maintain confidentiality of individual patients. This requirement to balance individual and relational privacy is one of the most challenging aspects of clinical practice in genetic medicine.

The impact of genetic interventions, such as predictive testing on the family, has been explored in a number of empirical studies, where the multi-layered dynamics between genetic knowledge or technology and family relations have been revealed. Some have examined the increasing awareness of the role of family history in assessing future health risks (Atkinson et al. 2006; Horstman and Finkler 2011), highlighted how common sense ideas of heredity inform understanding of genetics (Richards 1996) and how complex kin relations are thrown into relief by genetic interventions (Featherstone et al. 2006). Others have examined how genetic technologies have worked to re-establish the biological basis of kinship ties through the ‘medicalization of kinship’ (Finkler 2000). Yet, research on genetic interventions suggests that the social context and consequences of genetic information in the family and wider communities of social relations are diverse and dynamic (see, for instance, Horstman and Finkler 2011). For example, genetic testing for conditions such as familial hypercholesterolemia does not seem to entail genetic responsibilities to others, with many of those affected choosing not to inform unaffected family members about their risk (Weiner 2011). Yet, a quite different trajectory appears to play out in the context of conditions such as breast cancer and testing for the BRCA genes where gendered values are foregrounded in seeking care, not just for oneself but also for related others (Hallowell 1999 and Gibbon 2007).

In the context of cancer genetics, one of the most commonly given reasons for choosing to have a predictive test is to know about the risk for children (Lerman et al. 1995; Jacobsen et al. 1997). This is particularly marked in the case of breast cancer (Wakefield et al. 2011). It is notable that gender often plays a significant role in this reasoning, with the responsibility to share information about risk in the family often falling on women, positioned as kin ‘keepers and communicators’ of genetic information (Wilson et al. 2004) and as ‘selves in relation’ (Hallowell 1999). While the cultural value of female nurturance has been understood as a factor in the uptake and interest in predictive genetic testing (Gibbon 2007; see also Mozersky 2012), there is often a complex ethical tension or even a dilemma between an individual right to know, the right of others not to know and the potential consequences of risk information in the family (Gibbon 2007; see also Konrad 2005, for discussion of these dilemmas in relation to Huntington’s disease).

Increasingly, however, studies outside the Euro-American context are illuminating how specific notions of kin, family and citizenship have implications for the meaning and degree of engagement with genetic information (Horstman and Finkler 2011). This may be particularly relevant in a cultural context where the family traditionally play a key role in the management of health care and the sharing of health responsibilities.

## Empirical studies

In the final section of this paper, we draw on two different sources of empirical research within cancer genetic clinics in the south of Brazil. One relates to research carried out by the Bioethics Research Laboratory in Porto Alegre. The other is drawn from research undertaken in cancer genetic clinics in the south of Brazil, as part of Wellcome Trust funded project entitled “Admixture, Ancestry and Breast Cancer in Brazil: An Ethnographic Investigation of Population Genetics, Disease Risk and Identity”. This included participants’ observation in cancer genetic clinics over a period of 18 months in three different urban centres of Brazil, interviews with patients and family members attending cancer genetic clinics (n104) and interviews with practitioners and scientists working within or alongside cancer genetic specialists (n 41,. We draw from these two empirical data sets to further reflect on how patients and their families experience medical genetic services and also how obligations and responsibilities among family members inform the choices or have diverse consequences for those participating in genetic research.

## Cancer genetics, relational privacy and the family in southern Brazil

Similarly to studies outside Brazil focused on genetic testing for breast cancer for patients and research participants in the field of cancer genetics in Brazil, the question of care for others was prominent in the explanations about decisions to seek out genetic risk information. For many Brazilian patients, the logic of participating in genetic research related to a perceived ability to care for the family. As one patient (Cynthia)^1^ in São Paulo attending their cancer genetic clinics put it:

The doctor asked if I would like to participate. Logically I said yes, because my principal objective with this research is to help my family, in the sense of prevention. Now I want to know the possibilities for my daughter and for my nieces also.

For another patient, from Porto Alegre, this was not so much a choice, as an obligation reflected in the way she articulated her decision to participate in genetic research related to cancer risk in the family:

I have family, I have children, so you can’t not to do this.

Talking about the need to involve family members in genetic research, another patient, Celeste, from Sao Paulo talked more explicitly about the moral obligations this entailed for herself and related others.

You have to talk to everyone that they have to take care, everyone has to have prevention. I always say this. My sister asked ‘do you want to go’ [to the clinic]? I said ‘yes, I want to go’. Then, I grabbed my daughter. So, the first one came with me, and my sister and my niece. After that, we started to share this with others…, but not everyone wants to do the tests. It’s a very personal decision, but I still think it’s selfish not to do this. Because of your personal fear, you are losing the opportunity to have care; and, then, you discover your child needs it.

As these comments suggest, there is a sense of moral obligation to take care of the family, as participants in the cancer genetic clinics and in research. These sentiments about gendered responsibilities, expressed by those taking part in genetic testing for breast cancer, resonate with findings outlined elsewhere, in comparatively different national contexts (Gibbon 2007; Mozersky 2012; Kamprinai 2009). However, the strength of this articulation is particularly striking in Brazil. Here, the moral obligation to take care of the family is centrally situated in the motivation to participate in research. In Brazil, to choose not to participate is considered to be ‘selfish’.

Nevertheless, the experience of one participant from São Paulo, as outlined above, suggests the actual dissemination of genetic risk information in the family is uneven. We present here further research drawing on the methodological tool of the ‘sociogram’ (Moreno 1934) to illustrate how dissemination of information within the family is somewhat selective.

### The case of a family from south Brazil

We describe now one case study in detail, using the sociogram tool, to show how the shift from personal to relational privacy in cancer genetics is complexly configured by gendered ideas of responsibility and affective moral relations.

**Fig. 1:**
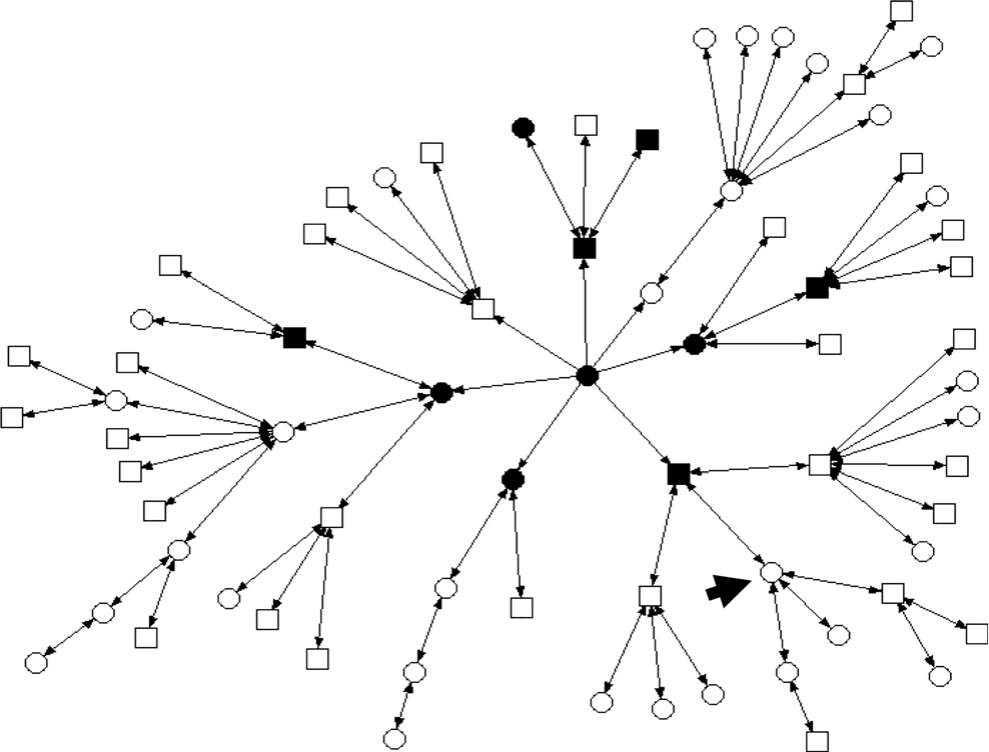
Sociogram of a family (72 persons) with one member diagnosed with a susceptibility to hereditary cancer. The *arrow* identifies the patient

The case concerns a patient who came to the cancer genetic clinics at the Hospital de Clínicas de Porto Alegre, south Brazil, for genetic counselling about susceptibility for hereditary cancer, after having had breast cancer herself. She told her oncologist that other family members, grandmother and father had died from cancer. After a preliminary evaluation of her family history and suitability for predictive genetic testing, the patient was tested and found to be carrier of a genetic mutation associated with increased risk for cancer. Exploring further with the patient the structure and background of her family, she revealed that there were 72 other members of her extended family, nine already diagnosed with cancer, in six different generations (see Fig. 1).

**Fig. 2:**
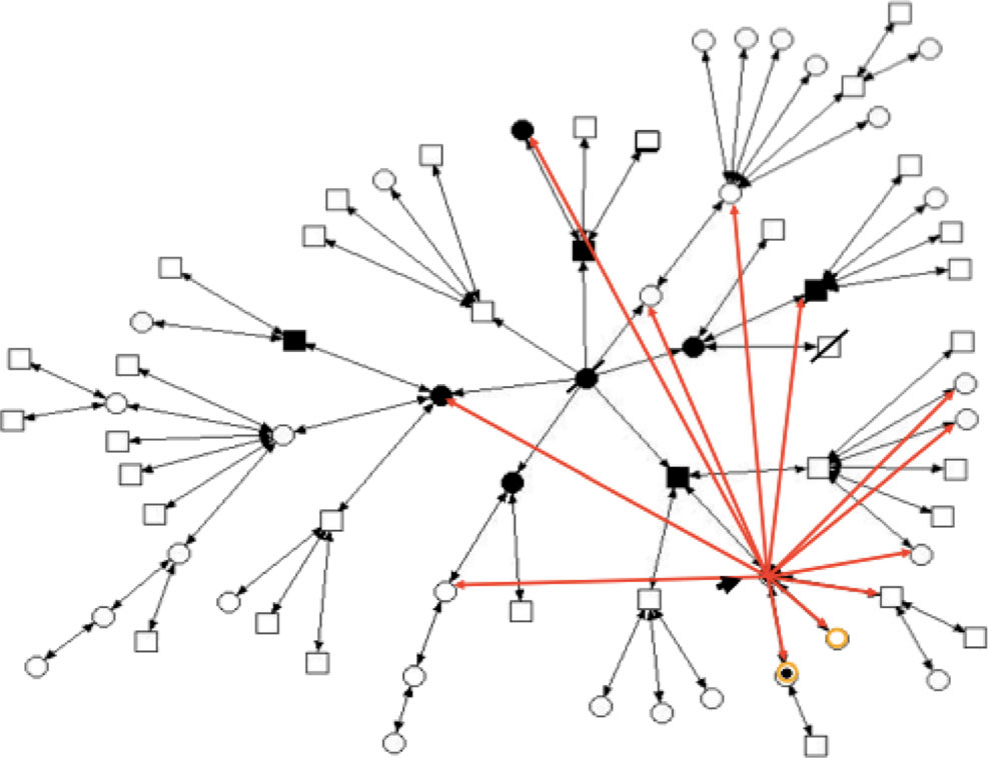
Sociogram with the first diffusion of information, performed by the patient, about the diagnosis of hereditary breast cancer susceptibility

**Fig. 3:**
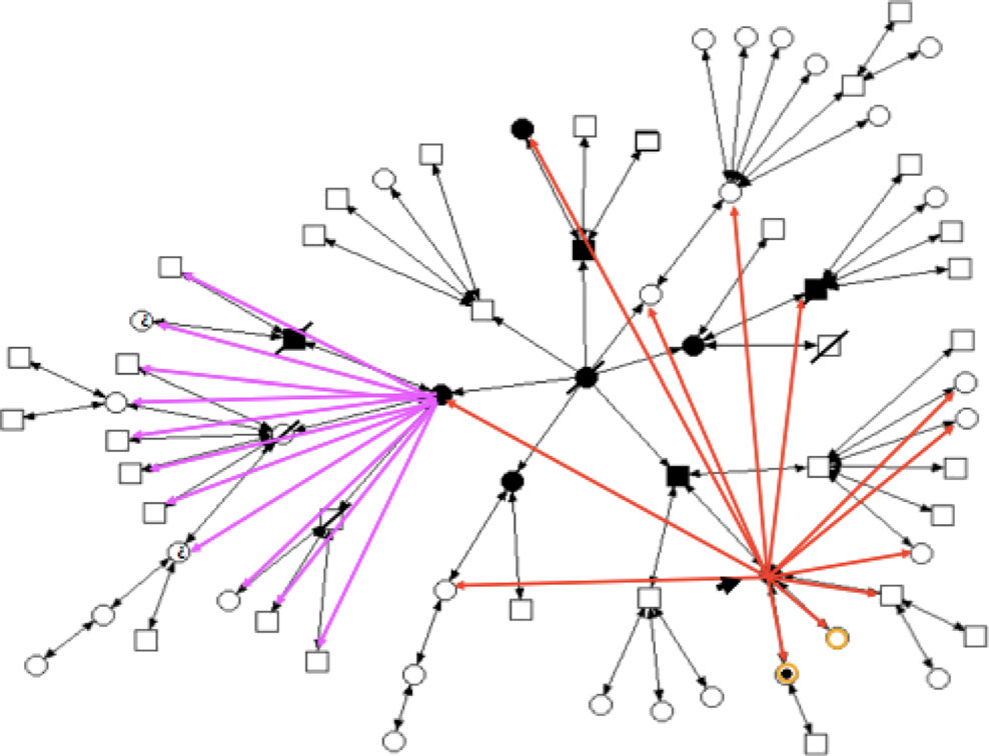
Sociogram with the second diffusion of information, by another relative of the patient, who also had a cancer diagnosis, about their susceptibility diagnosis for hereditary cancer

Of the five people who already died, three had cancer, one had cardiovascular problems and the other died in a car accident. The sociogram technique was used to track the way that information had and was being disseminated within the family. It revealed the patient had communicated this information to only 12 relatives: her three children, two aunts and seven cousins. One of these aunts had a diagnosis of cancer, while the other did not (see Fig. 2). The aunt who had had breast cancer, after being tested for the gene mutation, communicated that information to all her 11 grandchildren. It should be noted that this woman had three children, all already dead, of whom just one died from cancer (Fig. 3). Thus, from the initial identification of the genetic alteration in the first patient, 23 other people were informed of the situation, i.e. 34.3 % of family members.

It is notable, therefore, using a sociogram that sharing of information was selective. Two family branches were not informed at all of their potential risk. Another important aspect was that only two men were informed by the first patient: her son and a cousin, who had previously been diagnosed with throat cancer. This may be an example of selective relational privacy, because, although shared, the information was only partially disseminated within that family. Relational privacy is, thus, not simply the loss of personal privacy but a selective sharing with people who have some connection with the bearer of that information, according to the (understood) meaning of genetic risk within the family.

## Conclusion

Reflecting on the case of cancer genetics in Brazil, we attempted to show the necessity of addressing both personal and relational privacy in the process of informed consent.

The emphasis on autonomy in North American bioethics, which has served as the basis and justification for informed consent, has revealed the potential for an ‘empty ethics’ (Corrigan 2003). We suggest that Brazilian bioethics is formed by an alternative set of traditions (Goldim 2002), which shape how the challenges of privacy and informed consent are articulated and framed in new and emerging health fields, such as cancer genetics.

Drawing from examples and perspectives rooted in contrasting methodological and theoretical perspectives of both bioethics and anthropology, we try to illustrate what is at stake in the context of medical genetics in Brazil is both personal privacy and relational privacy. In this sense, the right to personal privacy, in terms of choosing not to undergo a genetic test or not share risk information, may not be the exercise of ‘selfishness’ but the desire to protect others in accordance with an understanding of the cultural meaning of genetic risk information and social relations in the family. At the same time, the necessarily complex interaction between health care and research in novel fields such as cancer genetics also informs these dynamics.

It is significant that new bioethical discussions in fields, such as medical genetics, concerning questions of privacy and consent are increasingly recognizing the importance of considering and incorporating questions not only of autonomy but also solidarity between professionals, patients, family members or members of a community (Schweitzer 1949; Ascensão 2006; Prainsack and Buyx 2011). These new approaches reflect an ongoing challenge and the necessity of understanding and facilitating informed consent as a process of continuous shared responsibility and deliberation.

## Footnotes

1 Note all real names have been changed and replace with pseudonyms
